# Partial Nephrectomy in pT3a Tumors Less Than 7 cm in Diameter Has a Superior Overall Survival Compared to Radical Nephrectomy

**DOI:** 10.7759/cureus.5781

**Published:** 2019-09-27

**Authors:** Brigitte K Ziegelmueller, Annabel Spek, Bernadett Szabados, Jozefina Casuscelli, Alexander Buchner, Christian Stief, Michael Staehler

**Affiliations:** 1 Department of Urology, University Hospital, Ludwig Maximilian University of Munich, Munich, DEU

**Keywords:** partial nephrectomy, overall survival, locally advanced disease, renal score, renal cell carcinoma

## Abstract

Objectives

We conducted this study to analyze the survival rates of patients with advanced renal tumors <7 cm in diameter treated surgically by partial nephrectomy (PN) compared to those who received radical nephrectomy (RN).

Material and methods

We retrospectively analyzed clinical data from 55 consecutive patients from our institutional database with T3a renal cell carcinoma of <7 cm treated surgically either by PN (*n *= 38) or RN (n = 17) in the Department of Urology of Ludwig Maximilians University from January 2006 to August 2014. The overall survival (OS) rates were calculated according to Kaplan-Meier estimation.

Results

The median age of the population was 67.9 years (range: 39.4 to 87.9 years). The median blood loss was 164.1 ml (range: 0 to 1200 ml), and the median clamping time was 8.85 minutes (range: 0 to 38 minutes). On average, the surgery lasted for 118 minutes (range: 40 to 210 minutes). The median serum creatinine level measured was 1.2 mg/dl (range: 0.7 to 2.3 mg/dl) preoperatively, and 1.4 mg/dl (range: 0.7 to 4.3 mg/dl) postoperatively. The median creatinine serum level measured during follow up was 1.4 ng/ml in individuals with a PN (range: 0.7 to 3.2 ng/ml), and 1.5 ng/ml in those with an RN (range: 0.9 to 4.3 ng/ml). Patients with an RN had a median OS of 38.6 months (range: 0 to 63.3 months). The median OS for patients with a PN was not reached after a follow-up of 80 months. The difference in OS in patients with PN and RN was statistically significant (*P *< 0.005).

Conclusion

Performing a PN in T3a tumors leads to better survival rates compared to an RN. In tumors <7cm, cT3a does not seem to be a contraindication for a PN. Further data should be analyzed to prove this survival benefit in a larger, multi-institutional cohort.

## Introduction

Surgery is carried out in a curative intention in patients with localized renal cell carcinoma (RCC). According to the European Association of Urology Guidelines, a partial nephrectomy (PN) is indicated in patients with T1a tumors (<4 cm). In T1b tumors (4 to 7 cm), a PN is favored over a radical nephrectomy (RN) whenever feasible. For T2 and advanced RCC, the decision to use PN should be made individually based on localization, kidney function, comorbidities, and surgical experience [1].

Nephron-sparing surgery improves overall survival (OS) in patients with localized RCC <4 cm, mainly due to the reduction in postsurgical kidney impairment [2-4]. A reduced glomerular filtration rate is associated with higher cardiovascular mortality, and an RN leads to a higher rate of kidney failure [2-4]. Several studies have shown a similar oncological outcome, a higher OS rate, and reduced morbidity after a PN [5-6]. Thus, PN has become the standard treatment for small renal tumors. Sometimes, after PN, the final pathology reports of small renal tumors reveal an advanced histological stage.

This study aimed to compare the oncological and functional results in patients who underwent a PN versus an RN with tumors classified as pT3a (<7 cm in diameter).

## Materials and methods

Patients and tumor assessment

This retrospective study included 55 consecutive patients from the Department of Urology, Ludwig-Maximilians University (LMU), Munich from 2006 to 2014 with renal tumors <7 cm in diameter who underwent either PN or RN and had a final pathological stage of pT3a.

Age, gender, presence of a solitary kidney, tumor-node-metastasis (TNM) stage, tumor size, type of surgery (PN vs. RN), blood loss, clamping time, complications, resection status, duration of surgery, Fuhrman grade, histologic subtype, preoperative and postoperative serum creatinine level, the Eastern Cooperative Oncology Group (ECOG) performance status, RENAL (radius, exophytic/endophytic properties, nearness of tumor to the collecting system or sinus in millimeters, anterior/posterior location relative to polar lines) nephrometry score, the presence of metastases, death, and local recurrence were assessed. The decision to perform PN or RN was based on preoperative clinical and imaging assessment. The tumor stage was determined according to the 2009 Union for International Cancer Control revised TNM classification [7]. 

Statistical analysis

The qualitative and quantitative variables were compared using the Chi-squared and Student’s *t*-test. OS rates were calculated with Kaplan-Meier analysis. Log-rank tests were used to compare differences between curves. P-values <0.05 were considered significant. All statistical analyses were processed with Statistical Package for the Social Sciences (SPSS) software version 17.0 (SPSS, Inc, Chicago, IL).

## Results

Patients, tumor characteristics, and surgical data

A total of 55 patients from the Department of Urology of LMU were included. There were 40 men (72.7%) and 15 women (27.3%). The median age at diagnosis was 67.9 years (range: 39.4 to 87.9), and the median tumor size measured 4.0 cm (range: 0.8 to 6.9 cm). A PN was performed in 38 cases, while an RN was performed in 17 cases. The ECOG performance status was zero in 47 patients (85.5%), one in seven cases (12.7%), and two in one patient (1.8%). The median RENAL nephrometry score was 2.3 (range: one to three), with five patients (9.1%) having a high score, 30 patients (54.5%) having an intermediate score, and 19 patients (34.5%) with a low RENAL score. The median blood loss was 164.1 ml (range: 0 to 1200 ml). The median duration of surgery was 118 minutes (range: 40 to 210 minutes). Complications occurred in one case (infection: 1.8%). The resection status showed R0 resections in 47 cases (85.5%), R1 in five patients (9.1%), and RX in one case (1.8%). Local recurrence was observed in four patients (7.3%). Distant metastases were observed in 14 cases (25.5%). Primary metastases were present in eight patients (14.5%). A secondary tumor was observed in one patient (1.8%). Ten patients (18.2%) died from their RCC, while 35 patients died from other reasons (63.3%) during the follow-up period of 40.1 months.

Comparison of patient and tumor characteristics according to the type of surgery

The duration of surgery was longer for an RN than for a PN (median duration: 3.02 vs. 1.54 hours, respectively). The tumor size was larger in patients that had an RN (mean: 4.4 cm; range: 0.8 to 6.9 cm) vs. a PN (mean: 3.9 cm; range: 1.0 to 6.5 cm). The amount of blood loss was comparable between RN and PN (170.6 ml vs. 161.8 ml, respectively). The median clamping time was 8.3 minutes for a PN. Complications occurred in one case of a PN and no cases of an RN. The median ECOG performance status was higher in those who received an RN compared to PN (0.2 vs. 0.1, respectively), but the RENAL score groups did not differ (7.3 vs. 7.3). There was a local recurrence that occurred in two cases in patients that received an RN and in two cases for those receiving a PN. A secondary tumor was revealed in one case of a PN within the contralateral kidney. The median serum creatinine level measured preoperatively was 1.2 mg/dl (range: 0.7 to 2.3 mg/dl); the median serum creatinine level postoperatively was 1.4 mg/dl (range: 0.7 to 4.3 mg/dl). The median serum creatinine levels during follow-up were 1.4 ng/ml in patients that received a PN (range: 0.7 to 3.2 ng/ml) and 1.5 ng/ml in those that received an RN (range: 0.9 to 4.3). There were no statistically significant differences observed between the two groups. Table 1 provides an overview of the patient characteristics of the two different groups.

**Table 1:** Clinicopathologic and baseline characteristics of the study population according to the surgical procedure (RN versus PN) ECOG, Eastern Cooperative Oncology Group; RCC, renal cell carcinoma; RN, radical nephrectomy; PN, partial nephrectomy; RENAL score, Radius/Exophytic/endophytic tumor location / nearness to the collecting system or renal sinus measured in millimeters (mm) as the shortest distance from the deepest point of the tumor, anterior or posterior location, location relative to the renal poles

Variables	RN	PN	Total
Number of patients	17 (30.9%)	38 (69.1%)	
Gender			40 men (72.7%)
			15 women (27.3%)
Mean age			67.9 years (39.4–87.9)
Mean follow up			80 months
Tumor size (cm)	4 (0.8–6.9)	3.9 (1.0–6.5)	4 (0.8–6.9)
Blood loss (ml)	170.6 (0–1200)	161.8 (0–500)	164.1 (0–1200)
Clamping time (min)		8.31 min (0–25)	
Complications	0	1	1 (1.8%)
Resection status			R0: n = 47 (85.5%)
			R1: n = 5 (9.1%)
			RX: n = 1 (1.8%)
ECOG	0.2	0.1	0: n = 47 (85.5%)
			1: n = 7 (12.7%)
			2: n = 1 (1.8%)
RENAL score	7.3	7.3	High: n = 5 (9.1%)
			Intermediate: n = 30 (54.5%)
			Low: n = 19 (34.5%)
Local recurrence	2	2	4 (7.3%)
Secondary tumor	0	1	1 (1.8%)
Metastases			14 (25.5%)
Death from RCC			10 (18.2%)
Preoperative creatinine level (mg/dl)	1.2 (0.8–2.3)	1.2 (0.7–2.2)	Not significant
Postoperative creatinine level (mg/dl)	1.5 (0.9–4,3)	1.4 (0.7–3.2)	Not significant

Comparison of OS according to the type of surgery

**Figure 1:**
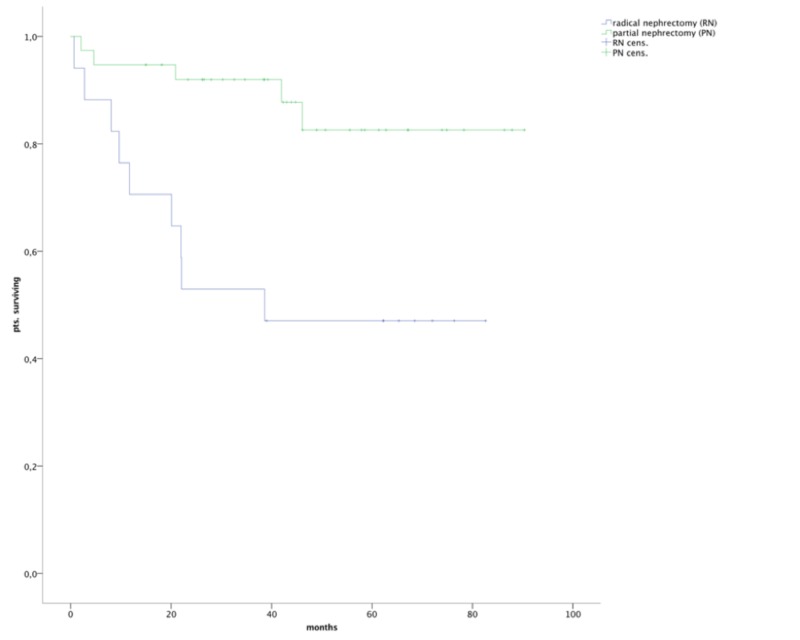
OS in patients after PN was higher than in patients after RN with statistical significance (p <0.005) OS, overall survival; RN, total nephrectomy; PN, partial nephrectomy; pts., patients

After a median follow-up of 80 months, the median OS after PN had not been reached and differed significantly from that after an RN, with a median OS after an RN of 38.6 months (range: 0 to 63.3 months; *P* < 0.005; Figure 1).

 

## Discussion

As the indications for a PN have been pushed towards larger tumors over the last decades, PN is conducted in even advanced renal tumors [6]. As a PN is more challenging than an RN, experienced surgical skills are necessary for these procedures. As a PN is conducted in patients with larger tumors, the number of incidental pT3a tumors rises and clinical to pathological T3a upstaging occurs more frequently [7]. 

Being confronted with such a pathology report, the urologist might wonder if an RN in pT3a RCC would have resulted in better cancer control. So far, there is no clear evidence on the oncological outcome in limited-size pT3a RCC treated by a PN. To our knowledge, we are the first to demonstrate that a PN in pT3a RCC <7 cm in diameter leads to a survival benefit compared to an RN.

Some studies investigated the outcome after PN in pT3a RCC. Jong Jin Oh et al. compared recurrence-free survival after PN (*n* = 45 patients) and RN (*n *= 298 patients) with clinical T1a, pathological T3a RCC and revealed a higher recurrence rate after RN during a 43-month follow-up (P< .001). In the PN cohort, there was no tumor size above 7 cm. The RN cohort included more large-sized tumors. Performing an RN in large pT3a tumors resulted in a higher risk of recurrence than for a PN in small pT3a tumors (*P *< 0.001); this could mean that large tumors might have additional aggressive features. This seems to indicate it may be best to not push the indication for a PN above the 7-cm cutoff in advanced renal tumors. In small renal masses (<4 cm), there was no significant difference in oncological outcome [8-11]. Thus, performing a PN in advanced small RCC seems to be possible. However, this study defined a cut-off of 4 cm.

Lee et al. evaluated recurrence-free, cancer-specific, and OS after PN in patients with cT3a pT3a RCC (*n* = 43) in comparison with those with pT1a lesions (*n *= 1342) and found similar oncological outcomes over a follow-up of 54 months (*P *= 0.521) [12]. There was no correlation to the outcome after an RN.

Another study evaluated recurrence-free survival after PN in patients with cT1 RCC upstaged to pathological T3a (134 patients of 1448) with a follow-up of 23 months. The recurrence-free survival was significantly lower in upstaged patients (76%) than in those not upstaged (93%; *P*< 0.001) [13]. The limitation of this study is the lack of a limit on tumor size for a PN to be indicated. The oncological differences between PN vs. RN are not addressed with these data.

Ramaswamy et al. demonstrated that pathological upstaging (66 patients, 44 with a PN and 22 with an RN) from cT1 to pT3a did not result in worsened oncologic outcomes after a follow-up period of 50 months [14].

Considering the outcomes of this study, the survival benefit for PN in patients with pT3a tumors <7cm, performing adjuvant therapy, considered in high-risk RCC, should be reevaluated. Ravaud et al. evaluated 615 patients with locoregional high-risk clear-cell RCC, who received adjuvant sunitinib therapy or placebo. The median disease-free survival was significantly longer in the sunitinib group than in the placebo group (6.8 years vs. 5.6 years; *P *= 0.03). As the OS is good in patients with small pT3a tumors after a PN, adjuvant therapy might not be useful in these patients and may be outweighed by the toxic side effects. Studies focusing on adjuvant therapy after a PN in patients with small pT3a tumors would not have any validity as these patients already have longer OS. Instead, the benefit of systemic adjuvant therapy after an RN in patients with pT3a tumors should be further evaluated as OS data are significantly worse and sunitinib as an adjuvant therapy has demonstrated longer disease-free survival [15].

## Conclusions

A PN in patients with pT3a tumors leads to prolonged survival rates compared to performing an RN in patients with tumors <7 cm. The cT3a status does not seem to be a contraindication for a PN. Further data should be analyzed to prove this survival benefit.
